# Biocompatibility, Bioactivity, and Antibacterial Behaviour of Cerium-Containing Bioglass^®^

**DOI:** 10.3390/nano12244479

**Published:** 2022-12-18

**Authors:** Sílvia R. Gavinho, Ana Sofia Pádua, Isabel Sá-Nogueira, Jorge C. Silva, João P. Borges, Luis C. Costa, Manuel Pedro F. Graça

**Affiliations:** 1 I3N and Physics Department, Aveiro University, 3810-193 Aveiro, Portugal; 2 I3N-CENIMAT, New University of Lisbon, 2825-097 Caparica, Portugal; 3 Associate Laboratory i4HB—Institute for Health and Bioeconomy, NOVA School of Science and Technology, NOVA University Lisbon, 2819-516 Caparica, Portugal; 4 UCIBIO—Applied Molecular Biosciences Unit, Department of Life Sciences, NOVA School of Science and Technology, NOVA University Lisbon, 2819-516 Caparica, Portugal

**Keywords:** Bioglass^®^, cerium, antibacterial properties, bioactivity, implant coatings

## Abstract

The main reason for the increased use of dental implants in clinical practice is associated with aesthetic parameters. Implants are also presented as the only technique that conserves and stimulates natural bone. However, there are several problems associated with infections, such as peri-implantitis. This disease reveals a progressive inflammatory action that affects the hard and soft tissues surrounding the implant, leading to implant loss. To prevent the onset of this disease, coating the implant with bioactive glasses has been suggested. In addition to its intrinsic function of promoting bone regeneration, it is also possible to insert therapeutic ions, such as cerium. Cerium has several advantages when the aim is to improve osseointegration and prevent infectious problems with dental implant placement. It promotes increased growth and the differentiation of osteoblasts, improves the mechanical properties of bone, and prevents bacterial adhesion and proliferation that may occur on the implant surface. This antibacterial effect is due to its ability to disrupt the cell wall and membrane of bacteria, thus interfering with vital metabolic functions such as respiration. In addition, its antioxidant effect reverses oxidative stress after implantation in bone. In this work, Bioglass 45S5 with CeO_2_ with different percentages (0.25, 0.5, 1, and 2 mol%) was developed by the melt-quenching method. The materials were analyzed in terms of morphological, structural, and biological (cytotoxicity, bioactivity, and antibacterial activity) properties. The addition of cerium did not promote structural changes to the bioactive glass, which shows no cytotoxicity for the Saos-2 cell line up to 25 mg/mL of extract concentration for all cerium contents. For the maximum cerium concentration (2 mol%) the bioactive glass shows an evident inhibitory effect for *Escherichia coli* and *Streptococcus mutans* bacteria. Furthermore, all samples showed the beginning of the deposition of a CaP-rich layer on the surface of the material after 24 h.

## 1. Introduction

The use of dental implants is increasing due to removable prosthesis limitations such as discomfort, no natural appearance, and the need for maintenance. However, the main reason for the growing market is aesthetic awareness [1]. Furthermore, dental implants are considered to be the only restorative technique that preserves and stimulates natural bone. Despite the impact of COVID-19 on the dental implants market, it is projected to grow globally to USD 4.12 billion by 2022 and is expected to achieve USD 6.34 billion by 2029, at a CAGR of 6.3% over the forecast period, 2022–2029 [2]. Despite the success of this practice, there are several risks associated with early and late dental implant failure. At the biological level, there is some early implant failure that can be related to host factors, such as systemic disease, implant design-related factors, and factors related to surgical trauma, for example, the damage of anatomical structures or an absence of primary implant stability. Late failures can occur and are typically due to destructive inflammatory processes such as peri-implantitis. This disease is a progressive inflammatory condition that affects the tissues surrounding an implant, leading to the loss of the supporting bone and implant failure. Peri-implantitis affects around 13% of implants and tends to increase by 0.4 to 43.9% within 3–5 years [3,4].

To minimize problems in the integration of the dental implant, other approaches are required such as implant coatings with bioactive glasses, due to their ability to promote bone regeneration [5]. The Bioglass^®^ (46.1SiO_2_–24.4Na_2_O–26.9CaO–2.6P_2_O_5_, mol%) proposed by Hench et al. has shown twice as much efficacy in the formation of new bone at the bone–implant interface than other biomaterials. This Bioglass forms bonds with the host bone, stimulating its formation due to its reaction mechanism that leads to the formation of a hydroxycabonate apatite layer on the glass surface with a mineral composition similar to that of bone [6,7,8].

Furthermore, it is possible to improve the biological response through the insertion into a bioactive glass network of therapeutic inorganic ions (i.e., zinc, copper, strontium, silver, and cerium) [9,10,11,12]. The use of cerium is becoming increasingly interesting due to its effects in protecting cells from ROS-induced damage, and reversing oxidative stress after implantation into bone, with a consequent enhancement of osteogenesis and reduction in bone healing time [13]. This catalytic activity as an ROS scavenger is due to the easy exchange of the oxidation states Ce^3+^ and Ce^4+^ [14]. This antioxidant effect for almost all noxious intracellular reactive oxygen species, which promote inflammation, is highly important in the surgical stress response [15]. Cerium promotes increased growth and the differentiation of osteoblasts, the mineralization of primary osteoblasts, increases collagen production in human mesenchymal cells, and improves the mechanical properties of bone [14,16]. Cerium also shows a fundamental and effective role in preventing bacterial adhesion and proliferation that may occur on the implant surface. Cerium ions bind rapidly to *E. coli* bacteria, interfering with respiration and other metabolic functions [14]. In addition, cerium-containing compounds used as antibiotics are also noted for their bacteriostatic ability, immunomodulation in degenerative pathologies, and antitumor agents [17]. For these reasons, and for showing excellent biocompatibility, cerium is used in the biological field such as bioanalysis, biomedicine, and drug delivery [15,18].

Thus, in this study, Bioglass 45S5 with several percentages of cerium was synthesized by the melt-quenching method as a potential multifunctional biomaterial to be used as a coating material for dental implants. Its morphological, structural, and biological properties were evaluated. The samples were tested for cytotoxicity, bioactivity, and antimicrobial activity to verify their suitability for use as dental implant coatings.

## 2. Materials and Methods

### 2.1. Synthesis Method

The base glass composition was synthesized considering the Bioglass^®^ developed by Hench et al. (46.1SiO_2_-24.4Na_2_O-26.9CaO-2.6P_2_O_5_, mol%) and 0.25, 0.5, 1, and 2 mol% of CeO_2_ were added to the glass, maintaining the molar composition of the Bioglass 45S5. The starting chemicals SiO_2_, P_2_O_5_, CaCO_3_, Na_2_CO_3_, and CeO_2_ (Merck KGaA, Darmstadt, Germany) were mixed and homogenized, using a planetary ball milling process for 1 h at 300 rpm. The mixed powder was calcined for 8 h at 800 °C. The melt-quenching process was performed in a platinum crucible at 1300 °C for 1 h. The different compositions were re-melted under the same conditions to improve the homogeneity of the glasses. The samples were studied in bulk and in a compressed powder form. For the powder samples, the bulk material was ground in an agate mortar to decrease the particle size and reduce the particle size distribution. For this, the powder was subjected to a grinding process in a planetary ball mill system, for 60 min at 300 rpm.

### 2.2. Morphological and Structural Characterization

The X-ray diffractograms (XRD) were obtained at room temperature on an Aeris-Panalytical (Malvern Panalytical B.V., Almelo, Netherlands) diffractometer, CuKα radiation (λ = 1.54056 Å) was generated at 40 kV, and 15 mA was used. The scanning parameters were: scan step of 0.002° at 38 s per step and a 2θ angle range between 10° and 60°.

FTIR spectra were acquired with a Bruker Tensor 27 FT-IR spectrometer (Bruker, Karlsruhe, Germany) in the range of 1100–400 cm^−1^ at a resolution of 4 cm^−1^ with 128 co-added scans, using pellets composed by KBr mixed with the powder of each sample in a weight ratio of 200:1 mg. During acquisition, the room temperature and humidity were kept at approximately 25 °C and 37%, respectively.

The sample surface morphology was evaluated by SEM (Scanning Electron Microscopy) using a microscope from TESCAN model Vega 3 (TESCAN ORSAY HOLDING, a.s., Brno – Kohoutovice, Czech Republic). A semi-quantitative examination of the chemical composition of the samples was performed using the Bruker EDS (energy dispersive spectroscopy) system coupled to the microscope. Several regions of each sample were analyzed using a square scanning area of 100 µm x 100 µm. All the sample surfaces were previously covered with carbon to enhance the surface electron conductivity.

### 2.3. Cytotoxicity Assay

The possible cytotoxic effects of the samples were assessed according to the “ISO 10993–5 Biological evaluation of medical devices—Part 5: Tests for in vitro cytotoxicity” standard using the extract method and the human osteosarcoma cell line (Saos-2 cells, ATCC^®^ HTB-85™). All powders were sterilized at 120 °C for 2 h. Both non-passivated and passivated extracts were produced at an initial concentration of 100 mg/mL. For non-passivated extract, the bioactive glass powder was incubated in McCoy 5A medium (Merck KGaA, Darmstadt, Germany) for 24 h at 37 °C in the culture medium. After incubation, the extract was filtered with a millipore filter with 0.22 µm and stored at 37 °C. For the passivated extract, the same bioactive glass powder was incubated for another 24 h at 37 °C in incubated McCoy 5A medium.

The Saos-2 cell lines were seeded in 96-well plates and incubated at 37 °C with 5% CO_2_ for 24 h. The culture medium was removed and replaced with non-passivated and passivated extracts with successive dilutions (50 mg/mL, 25 mg/mL, 12.5 mg/mL). A positive control was formed using cells in a cytotoxic environment created by the addition of 10% dimethyl sulphoxide (DMSO), and negative controls were viable cells.

After 48 h of the cell culture being in contact with the extracts, a colorimetric viability assay using resazurin was performed. The reduction of the oxidized non-fluorescent blue resazurin to a red dye (resorufin) is due to the mitochondrial respiratory chain in live cells. The solution of resazurin and medium 1:1 was reacted for 3 h, and the absorption at wavelengths of 560 nm and 600 nm was measured [19].

To verify the reproducibility of the assay, three biological replicates were performed with six statistical replicates in each.

### 2.4. Bioactivity

The bioactivity assay was assessed in pellets with 7 mm of diameter, pressed for 5 min at 2 tones. According to “ISO 23317—Implants for surgery—In vitro evaluation for the apatite-forming ability of implant materials” standard, the bioactivity was analyzed. The samples were immersed in simulated body fluid (SBF) under stirring, removed from the medium, and cleaned with deionized water after 12 h, 24 h, 48 h, 96 h, 336 h, and 672 h. On one set of samples, every two days, the medium was replaced to mimic the biological environment as much as possible. The surface of the samples was analyzed by SEM-EDS. In addition, the pH of the SBF medium was measured both in the set of samples in which the medium was changed and in the samples where the medium was not changed at all. The assay was performed in duplicate.

### 2.5. Antibacterial Activity

To detect the antimicrobial behaviour of the several compositions of Ce-containing Bioglass, the method of agar diffusion assay plates was used with the reference strains *Escherichia coli* K12 DSM498, *Staphylococcus aureus* COL MRSA (methicillin-resistant strain), and *Streptococcus mutans* DSM20523. The bacterial strains were cultivated in tryptic soy broth (TSB) at 37 °C overnight. The pellets with 7 mm of diameter were previously sterilized at 180 °C for 2 h.

The two-layer bioassay was performed using the TSB solidified with agar 1.5% *w*/*v*, base layer, and 0.8% *w*/*v*, top layer. Plates were prepared with an 18–20 mL base layer and 4 mL of molten seeded overlay containing approximately 10^8^ CFU/mL of the appropriate indicator bacteria. At the center of the plate, the disks of material to be tested were deposited, and the plates were incubated for 24 h at 37 °C. For *S. mutans*, an incubator maintained at 5% CO_2_ was used.

Images of the pellets were taken, and the diameters of the inhibition halos were measured with ImageJ software; each pellet was measured 50 times in various orientations [20]. The data of the three independent assays were statistically analyzed with an unpaired t-test, comparing the bioactive glass base composition with each of the different samples using GraphPad Prism 8.0 software.

## 3. Results and Discussion

**Figure 1:**
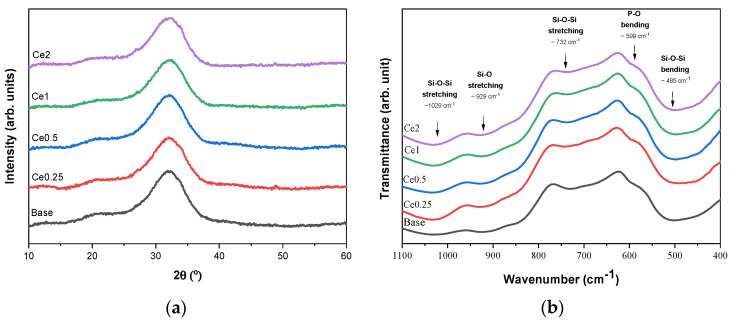
(**a**) XRD diffractogram and (**b**) FTIR spectra of Bioglass 45S5 with several concentrations of cerium (0, 0.25, 0.5, 1, and 2 mol%).

Figure 1a presents the XRD diffractogram showing a broad band in the range of 25–38° 2ϴ for all samples, characteristic of an amorphous phase [10,21]. The XRD data demonstrates that the addition of the cerium ions does not modify the glass network at the structural level, even at the highest cerium concentration [22]. As dissolution and ion release kinetics depend on the structure of the glass network and the type of ions present in the glass, it is crucial to maintain the structure of the glass matrix without compromising the bioactivity [23].

Figure 1b shows the FTIR spectra of Bioglass^®^ with the different concentrations of cerium. In agreement with the XRD results, the characteristic absorption bands of the amorphous bioactive glass can be observed for all samples, indicating no modification of the glass matrix with the addition of cerium up to 2 mol%. The bands around 1029 cm^−1^ and 929 cm^−1^ are associated with the Si-O-Si asymmetric stretching mode, 732 cm^−1^ is assigned to the Si-O-Si symmetric stretching mode, and 485 cm^−1^ is associated with the Si-O-Si bending mode. The shoulder at 597 cm^−1^ is related to the P-O bending mode from amorphous phosphate observed for Bioglass 45S5 [24,25,26,27,28].

**Figure 2:**
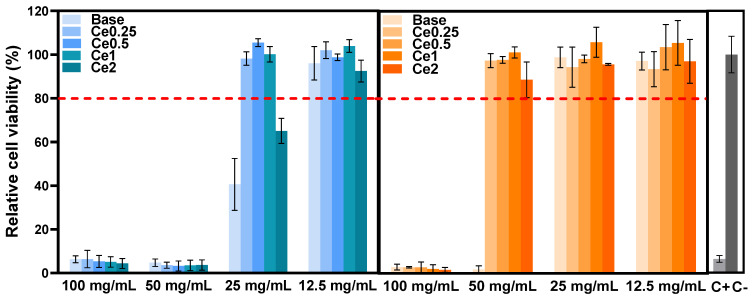
Relative cell viability of Saos-2 cells after 48 h incubation with glass extracts (**left** side: non-passivated extracts; **right** side: passivated extracts).

The Saos-2 cell line viability in contact with glass extracts is shown in Figure 2. The effect of extract contact with the cell line on proliferation was measured by resazurin assay to determine whether the compositions studied can be safely applied in bone regeneration. The results show that extracts that were not preconditioned with McCoy’s culture medium (passivated extracts) show a severe level of cytotoxicity (cell viability below 10%) at the concentration of 100 mg/mL and 50 mg/mL. At a dilution of the extract of 25 mg/mL, the cytotoxicity to the cells decreases, and in the samples containing cerium, the cell viability is higher compared to the base bioactive glass sample (left side graph). It is therefore observed that the addition of CeO_2_ to the base bioactive glass can increase the biocompatibility of the materials, which is in accordance with the findings of other studies [15,16,17,29].

Furthermore, the cytotoxicity of the extracts decreased when the materials were subjected to a passivation process (right side graph). The cell viability results for the passivated extracts show non-cytotoxicity for the samples with CeO_2_ at extract concentrations of 50 mg/mL, and the base also does not show cytotoxicity at 25 mg/mL. Comparing the results of passivated and non-passivated extracts, it is important to consider that cytotoxicity is associated with an increase in local pH due to a high rate of ion-exchange reactions that occur when the material interacts with the cell culture medium during the first 24 h [27]. When the bioactive glass comes into contact with the cell medium, there is a breakdown of the Si-O-Si bonds, and the release of soluble silica into the solution in the form of Si(OH)_4_ occurs. The rate of dissolution increases the pH of the surrounding environment, which affects metabolism and cellular function and may even influence gene expression. Furthermore, osteoblasts are extremely sensitive to extracellular pH in in vitro tests, increasing the efficacy of osteoblastic activity and cell proliferation at near-neutral pH (7.0–7.6), with downregulation at pH 7.8. Moreover, the function of osteoclasts is indispensable in the initial phase of bone regeneration, promoted by acidic environments. However, this effect is not visible in the in vivo environment due to the existence of dynamics that allow the organism to regulate and balance the pH. Thus, in vitro studies to assess cell viability have been defined in order to limit pH changes that are not present in vivo [30].

**Figure 3:**
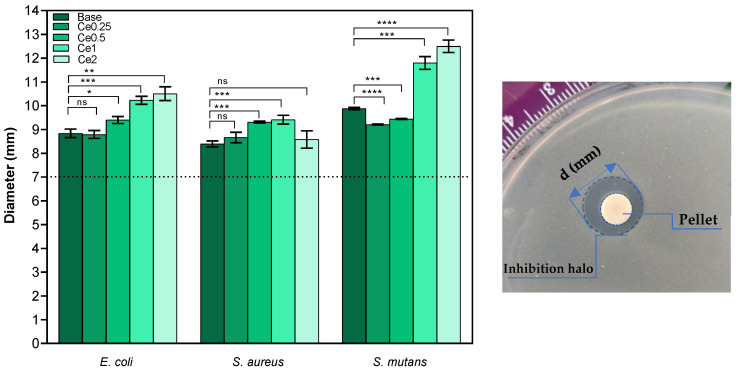
Measurements of inhibition halo diameter of all samples against Gram-negative (*E. coli*) and Gram-positive (*S. aureus* and *S. mutans*) bacteria, 24 h after incubation (ns: nonsignificant; * *p* ≤ 0.1; ** *p* ≤ 0.01; *** *p* ≤ 0.001; **** *p* ≤ 0.0001). **Left** side: Means of inhibition halo diameter for all microorganisms. **Right** side: example of a plate assay showing the inhibition halo of Ce2 pellet on *S. mutans*.

The evaluation of the antimicrobial activity was analyzed by the agar diffusion method for *E. coli* and *S. aureus* as models of Gram-negative and Gram-positive bacteria, respectively. Furthermore, the effect of the bioactive glasses against *S. mutans* was also determined due to its direct relationship with the presence of oral biofilms and dental caries. The results reveal that all materials show antibacterial activity against all bacterial strains as they all show a halo of inhibition with mean values higher than 8.39 mm (Figure 3). In the case of *E. coli*, the samples that present higher mean values of diameter are samples Ce1 and Ce2 with mean values between 10.23 mm and 10.51 mm, respectively. For *S. aureus*, the average diameter values are lower than those observed for the other bacteria, and no significant effect is noticed in samples Ce2. However, sample Ce1 presents the highest antibacterial effect against S. aureus with an average value of 9.41 mm. On the other hand, the antibacterial effect is more evident against S. *mutans* (Gram-positive) in samples Ce1 and Ce2 with mean diameter values between 11.8 mm and 12.5 mm, respectively. Studies have already revealed that CeO_2_ shows less or no antibacterial activity against S. *aureus* and that bioactive glass scaffolds with cerium also did not exhibit antibacterial activity [18,31]. Conversely, other studies reported antibacterial activity towards S. *aureus* [[16] and references therein]. Nevertheless, in general, increasing amounts of Ce correlate with a higher antibacterial effect against the set of bacteria evaluated, suggesting that the antibacterial effect is associated with the presence of cerium ions. Although researchers present studies confirming the antibacterial effect of cerium oxide, not all mechanisms related to the action of cerium in killing bacteria are yet clarified. Many mechanisms of action are suggested as the facility of Ce^3+^ to penetrate the cytoplasm of cells and interfere with endogenous respiration and interact with phosphate compounds and proteins. Furthermore, the facilitated exchange of Ce^3+^ and Ce^4+^ oxidation states suggests oxidative stress exerting cytotoxicity to bacteria [32].

Antibacterial activity was also demonstrated for the bioactive glass base sample, and the mechanisms underlying its bactericidal effect are still elusive [[16] and references therein]. Several mechanisms may be associated with this effect, namely, changes in pH in the medium to alkaline values and osmotic pressure. As mentioned above, the dissolution of ions into the medium promotes a pH increase, which becomes stressful for bacteria, leading to their death. In this study, the pH measured in the TSB medium after 24 h incubation of base and Ce2 pellets reached ≈ 11. Furthermore, the release of the various ions affects the membrane potential in the bacteria and causes a higher osmotic pressure. Generally, the concentration of solutes is higher inside the bacteria than in the external environment, resulting in positive pressure on the cell membrane. When there is indeed an increase in external solutes due to the dissolution of Bioglass^®^ to the surrounding environment, it causes a rapid efflux of water and a pressure drop across the cell membrane, modifying the cell size, shape, and membrane tension levels [33]. However, this in vivo effect is not verified due to the buffering system present in the organism, and thus the presence of antimicrobial agents is important for the antimicrobial effect to remain.

**Figure 4:**
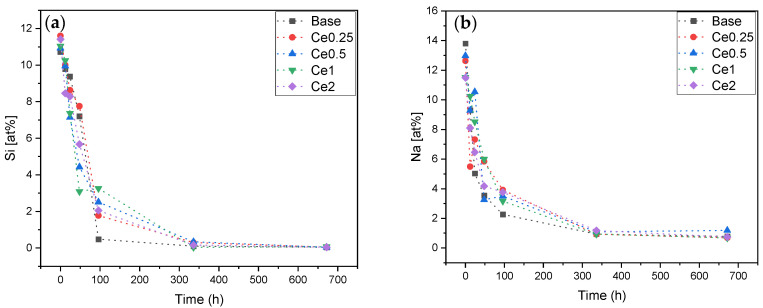

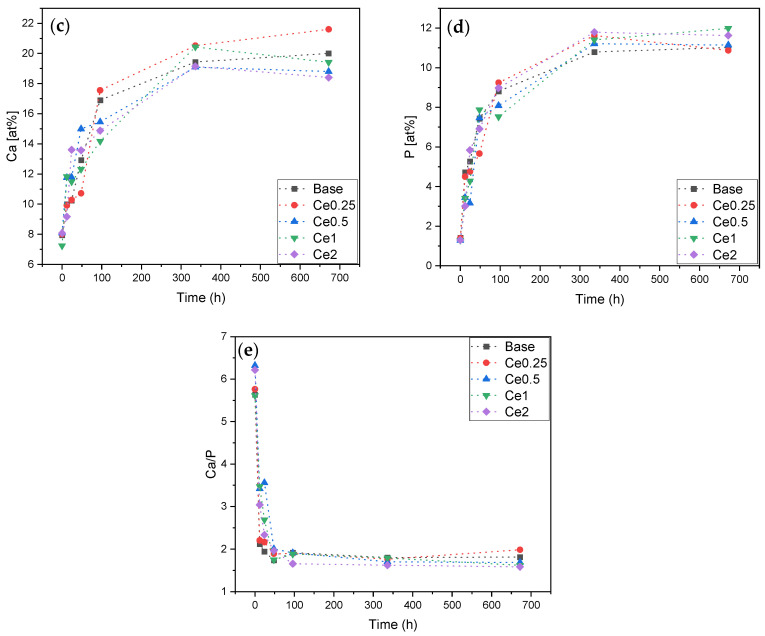
EDS of pellets surface after SBF immersion (12 h, 24 h, 48 h, 96 h, 336 h, and 672 h). (**a**) Si at%; (**b**) Na at%; (**c**) Ca at%; (**d**) P at%; and (**e**) Ca/P ratio.

In order to estimate the ability of new bone formation and the bonding of the materials to the host bone when inserted in the body, the formation of the apatite layer produced when the bioactive glass is immersed in SBF was evaluated. The pellets were immersed in SBF according to “ISO 23317:2014 Implants for surgery—In vitro evaluation for the apatite-forming ability of implant materials” standard for 12 h, 24 h, 48 h, 96 h, 336 h, and 672 h, and their surfaces were evaluated by SEM-EDS in order to quantify the atomic % of each ion (Si, Na, P, and Ca) on the pellets’ surfaces for the different times immersed (Figure 4).

The reaction mechanism when the bioactive glass is immersed in SBF starts with an ionic exchange between the sample and the surrounding medium and a rapid release of soluble ionic species occurs. During the first 24 h, a layer of Si–OH is formed on the surface, leading to an increase in pH and consequently the formation of soluble Si(OH)_4_. The formation of the silica gel layer allows the absorption of ions from the environment, and there is also diffusion of Ca^2+^ and phosphate (PO_4_^3−^) through this layer to the sample surface forming an amorphous calcium phosphate film which will subsequently crystallize [6,9,34,35]. Figure 4a,b shows a decrease in the amount of Si and Na at the sample surface over time due to the formation of the amorphous Ca and P layer and the dissolution of Si and Na into the surrounding medium. This result is verified in other studies that evaluate the amount of Si released into the medium as that it tends, contrarily to what is measured on the surface of the pellets, to increase during the first 4 days, stabilizing in the following days [29]. The formation of the calcium and phosphorus-rich layer is verified with the increase in the at% of both elements at the sample surface over the immersion time as shown in graphs 4(c) and 4(d). The Ca/P ratio suggests the formation of the apatite layer, as its value approaches the Ca/P ratio of hydroxyapatite (Ca/P ≈ 1.67) [36]. The sample that most rapidly approaches this value is the Ce2 sample (Ca/P = 1.66) at 96 h, indicating that the insertion of CeO_2_ does not influence negatively the bioactivity of the Bioglass^®^ [29].

**Figure 5:**
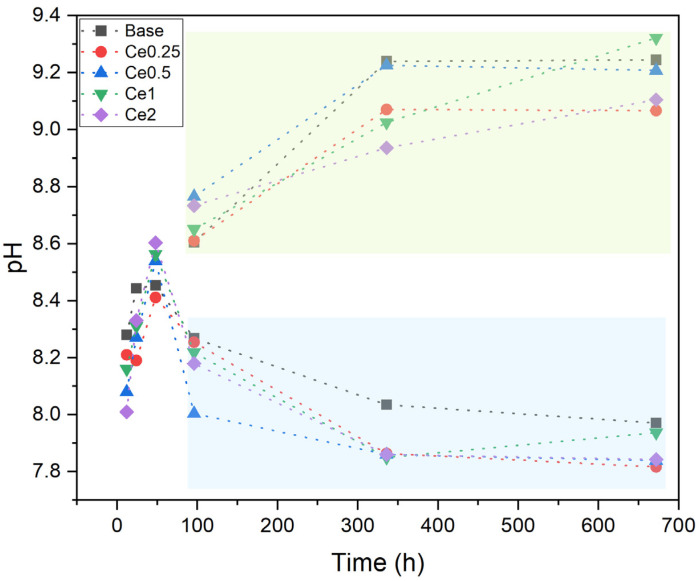
pH values of SBF medium after immersion of all samples in EDS for 12 h, 24 h, 48 h, 96 h, 336 h, and 672 h with medium change every two days (blue rectangle) and without medium change (green rectangle).

The pH of the SBF was also measured at all time points for all samples (with and without medium change every two days) as shown in Figure 5. As previously discussed, the pH of the solution surrounding the sample tends to increase over the immersion time, being its rise, up to a maximum of ≈9.3, more evident during the first 48h and slower during the remaining period as verified by the values surrounded by the blue rectangle (samples for which the medium was not changed). However, simulating what happens in the organism with the medium change every two days, it was verified that the pH tended to lower values becoming constant after 96 h. This pH decrease is attributed to the formation of the apatite layer on the surface of the bioactive glasses [29,37].

**Figure 6:**
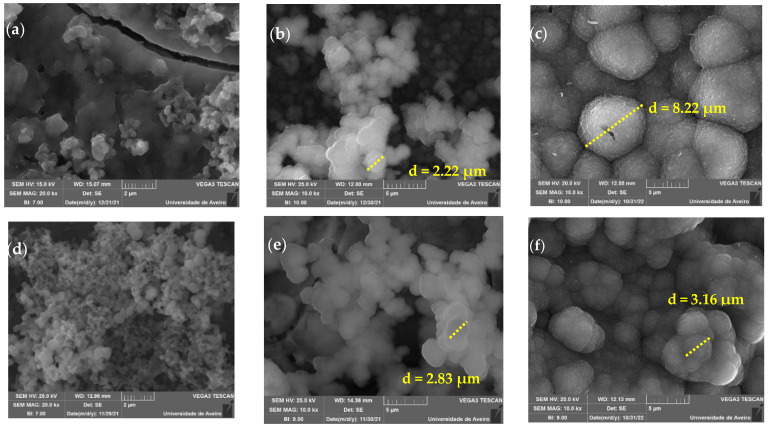
SEM images of glass pellets after 24 h, 96 h, and 336 h of immersion in SBF (**a**) Base 24 h; (**b**) Base 96 h; (**c**) Base 336 h; (**d**) Ce2 24 h; (**e**) Ce2 96 h and (**f**) Ce2 336 h.

Figure 6 shows the SEM micrographs taken at the pellet surface after 24 h, 96 h, and 336 h of the base and Ce2 samples. The growth of a layer of spherical particles that tend to increase in size with immersion time is visible in both samples. The base bioactive glass sample for 24 h of immersion presents particles with sizes in the range of 200–400 nm, and the sample with 2 mol% cerium shows particles with a larger size distribution between 100–700 nm for the same immersion time. After 96 h, the Ca and P layer present at the surface of the samples becomes more complete, and there is a substantial increase in the size of the particles, with sizes of approximately 2 µm. At 336 h, the micrographs show the surface fully coated by the precipitated apatite layer showing cauliflower morphology and with particles of the order of 8 µm, which suggests the deposition of a bone-like layer. The images indicate that the cerium-containing bioactive glass has potential as an osteoconductive material [38].

## 4. Conclusions

The results show that cerium-containing bioactive glasses synthesized did not present changes at the structural level for concentrations up to 2%mol of CeO_2_. Additionally, the addition of cerium has a positive effect on biocompatibility, decreasing the toxicity of Bioglass^®^. Furthermore, the results of all passivated samples with cerium show decreased cytotoxicity and are no longer toxic at 50 mg/mL. The 2%mol bioactive glass was also the one with the highest antibacterial activity, and the bioactivity characteristic of the Bioglass^®^ was not compromised by the addition of CeO_2_.

Thus, the Ce2 bioactive glass presents potential applications in bone regeneration, the filling of bone defects, or as implant coating.

## Data Availability

Not applicable.
